# Supplemental enteral tube feeding nutrition after hospital discharge of esophageal cancer patients who have undergone esophagectomy

**DOI:** 10.1007/s10388-020-00803-z

**Published:** 2021-01-21

**Authors:** Masahiro Niihara, Yasuhiro Tsubosa, Aiko Yamashita, Keita Mori, Hiromi Tsumaki, Yusuke Onozawa, Hiroyuki Fukuda

**Affiliations:** 1 Shizuoka Cancer Center, Division of Esophageal Surgery, 1007 Shimonagakubo, Nagaizumi-cho, Sunto-gun, Shizuoka 411-8777 Japan; 2 Shizuoka Cancer Center, Nutrition Support Team, Sunto-gun, Japan; 3 Shizuoka Cancer Center, Clinical Trial Coordination Office, Sunto-gun, Japan; 4 Department of Upper Gastrointestinal Surgery, Kitasato University School of Medicine, Sagamihara, Japan

**Keywords:** Esophageal cancer, Home enteral nutrition, Intermittent feeding

## Abstract

**Background:**

After undergoing esophagectomy to treat esophageal cancer, there are changes in the normal intake patterns in most patients, with more than half found to have an inadequate oral intake at the time of their hospital discharge. However, the use of home supplemental enteral tube feeding nutrition after hospital discharge in esophagectomy patients has yet to be established. The aim of this study was to evaluate the feasibility of 90-day home supplemental enteral tube feeding nutrition in esophagectomy patients.

**Methods:**

This single-center, prospective, and single-arm study evaluated the feasibility of using supplemental tube feeding nutrition intervention for 90 days in esophageal cancer patients who have undergone esophagectomy.

**Results:**

This study enrolled 24 post-esophagectomy patients between February 2015 and September 2016. Twenty patients were administered 70% or more of the planned nutrient, with 83% of the patients completing the nutritional intervention procedure. There were no grade 3/4 adverse events observed, with a mean body weight change of − 7.6 ± 6.0%.

**Conclusions:**

Our results showed that routine use of 90-day home supplemental enteral tube feeding nutrition after hospital discharge for esophagectomy patients was both feasible and acceptable.

**Trial registration:**

UMIN000016286.

## Introduction

Mortality from esophageal cancer in Japan accounts for 3.45% of all deaths from all malignant neoplasms, making it the seventh most common type of cancer among Japanese men. In recent years, there has been a rise in these morbidity rates. The peak age for the disease is at 60 years, with 46% of these patients aged 70 and older, which is a relatively large number of elderly subjects. Among these patients, the frequency of cStage I esophageal cancer is approximately 20%, while cStage II/III (except cT4) accounts for about 50%, with all of these subjects candidates for esophagectomy [1].

After undergoing esophagectomy, there can be changes in the normal intake patterns due to complications such as asthenia, pain, anorexia, and disorders in the digestion processes. Studies have shown that over 60% of these patients have an inadequate oral intake at the time of hospital discharge, only meeting 70% and 65% of their energy and protein requirements, respectively [2].

It has been reported that patients require 3–9 months to regain a defined eating pattern after the esophagectomy. During 6 months after the procedure, most of these patients lose more than 10–15% of their body mass index (BMI), and are, therefore, at severe nutritional risk, which can negatively affect their quality of life (QOL). Moreover, esophageal resection has been reported to have a negative impact on the global, functional, and symptom health-related QOL scores at 3 months [3].

Enteral nutrition started within 48 h might potentially have a positive impact on the clinical outcomes during the immediate postoperative period. Early enteral nutrition is safe, economic, and superior for reduction of postoperative complications, in addition to promoting recovery of intestinal movement, and early recovery from systemic inflammation [4]. However, enteral nutrition is often discontinued at the start of the oral intake and discharge from the hospital.

Nutritional support is considered important throughout the early postoperative period, and for the medium- and long-term periods after hospital discharge. As mentioned above, it is known that the oral intake decrease after esophagectomy continues for a period of time after the discharge, thus making it difficult to secure sufficient nutrition even if oral nutritional supplements (ONS) are used. In particular, nutritional intervention for up to 3 months after the surgery, which is the time when weight loss is at its greatest and the health-related QOL is at its lowest, is important. According to the European Society of Parenteral and Enteral Nutrition (ESPEN) guidelines, it is strongly recommended that “Supplemental enteral tube feeding is given to patients whose oral intake of food and fluids is inadequate for reaching their defined target alone” [5]. However, the practice of home supplemental enteral tube feeding after hospital discharge for esophagectomy patients has not been established.

Generally, jejunostomy and gastrostomy tubes, which are created at the time of the esophagectomy, are considered to be good for continuous administration. This is because the tip of the tube is located in the upper jejunum, as the administration of nutrients after the pylorus along with intermittent feeding is not recommended. However, continuous administration requires an infusion pump (electronic feeding pump), which may cause problems with regard to daily routines and reintegration into previous activities, such as going out and/or getting back to work. Therefore, we divided up the planned daily dose per day and then administered it using the Catheter Tip Syringes without the assistance of an infusion pump. This intermittent tube feeding nutrition after discharge is referred to as supplemental enteral tube feeding nutrition (STN). This procedure is not expected to affect the oral intake of food and fluids to any great degree, as it does not compete for the limited gastric tube volume. At the same time, however, there have been a few reports regarding whether it is practical to administer scheduled doses due to the time and effort required for the education of patients and families, and whether or not intermittent feeding is actually possible from a practical basis.

The aim of our study was to evaluate the feasibility of using the 90-day STN at home in esophagectomy patients after hospital discharge.

## Materials and methods

### Study overview

This single-center, prospective, and single-arm study evaluated the feasibility of STN intervention for 90 days in esophageal cancer patients who had previously undergone esophagectomy.

This clinical trial was registered on the University Hospital Medical Information Network (UMIN) Clinical Trial Registry website (UMIN000016286). The study was approved by the Institutional Review Board of the Shizuoka Cancer Center in December 2014.

### Participants

This study enrolled 24 patients.

#### Patient inclusion criteria

(a) Age > 20 years, < 75 years.

(b) Histologically diagnosed esophageal cancer.

(c) Clinical stage I, II, III, IV (UICC 7th).

(d) Curative resection (R0).

(e) Subjects underwent planned elective esophagectomy and retrosternal gastric tube reconstruction with cervical anastomosis.

(f) Placement and implementation of a feeding catheter in the jejunum trans-gastric tube during the surgical procedure.

(g) Home discharge.

(h) Life expectancy > 6 months.

(i) No preoperative treatment or only neoadjuvant treatment.

(j) Written informed consent provided by the patient.

#### Patient exclusion criteria

- Clinically T4b.
- Metastasis of the distal organ without supraclavicular lymph node metastasis.
- Impossible to perform nutrition intervention.
- Subject was pregnant or a woman of child bearing potential.
- Ineligible for this clinical trial, as determined by the attending physician.

### Intervention

Patients and their family (if possible) were taught to independently manage the STN at home. For the first 90 days after discharge from the hospital, participants administered the STN during the daytime using Catheter Tip Syringes. The planned daily STN dose consisted of the administration of one Enevo™ 250 mL can (1.2 kcal / mL) (Abbott). The 250 mL planned daily dose was divided into 4 or 5 intermittent feeding doses of 50 to 60 mL each.

Both increases or decreases in the total dose and increases or decreases in the total number of doses were considered acceptable. In our institution, the criteria of postoperative hospital discharge were that the oral intake was 60% or more of the required energy. In oral diet cases, nutritional guidance was provided when necessary, with instructions given to the patients on how to avoid reducing the oral intake of the enteral nutritional supplement administration as much as possible.

### Outcome measures

The primary outcome measures in this pilot study were chosen to determine whether or not the planned home supplemental enteral tube feeding nutrition would be feasible.

The total planned dose for the whole period was 22,500 mL (27,000 kcal) in 90 cans of Enevo™. A treatment for 90 days after the discharge from the hospital was defined as being complete if there was enteral administration of 15,750 mL (18,900 kcal), which was 70% of the planned dose. We confirmed the administration situation at the time of the outpatient visits that occurred every 2 weeks. After any temporary interruption, results were considered acceptable if the patient was able to resume the administration and achieve the required dosage. However, the administration of more than one 250 mL can of Enevo™ per day was not acceptable.

Additional measures to be recorded at 3 months and 6 months after baseline focus on the nutritional status and skeletal muscle volume, specifically nutritional parameters of body weight, body mass index, serum albumin and serum transthyretin (prealbumin) as secondary factors.

### Statistical analysis

The primary endpoint of this study was the treatment completion rate. At the present time, STN at home after esophagectomy is not routinely carried out, and there have yet to be any published data on the variables of interest in this patient population. For the contiguous sample size in this study, the threshold completion rate was 60%, the expected treatment completion rate was 85%, the two-sided significance level was 10%, the detection power for this analysis was 85%, and there were a total of 24 cases evaluated [6]. For the primary endpoint analysis, the point estimation and interval estimation were performed. Point estimates were calculated using the total number of cases registered and the number of cases successfully treated. Interval estimation was calculated by the method of constructing an accurate confidence interval based on the binomial distribution. If the 90% confidence interval (90% CI) for the treatment completion rate exceeded the set threshold completion rate (60%), it was decided that the study was feasible.

The secondary endpoints were the changes of body weight, skeletal muscle in a cross-sectional area (CSA) at the third lumbar vertebra (previously described [7]), and the type and frequency of the adverse events (AEs). A safety analysis was conducted in all of the patients who received STN at least one time, and AEs were assessed and graded using Common Terminology Criteria for Adverse Events (CTCAE) v4.0. The data were analyzed using the SPSS 21.0 Software (IBM Corp., Armonk, NY, USA). Results are expressed as mean ± standard division (SD) for quantitative variables. The Shapiro–Wilk test was used as a statistical method for normality. The values of median, 25th percentile and 75th percentile were described in the Boxplots.

## Results

### Baseline characteristics

Between February 2015 and September 2016, 24 esophagectomy patients were enrolled in this study. All participants were included in the safety analysis and the full analysis set. Table 1 summarizes the patient characteristics. There were 18 males and 6 females, with a mean age of 61.8 ± 9.4 years. Mean body weight were 57.3 ± 11.6 kg. Among the participants, 7 (29.2%) had stage I disease, 4 (16.7%) had stage II disease, and 13 (54.2%) had stage III disease.

**Table 1:** Patient characteristics

	*n* = 24
Sex	
Male	18
Female	6
Age in years^a^	61.8 ± 9.4
Histology	
Squamous cell carcinoma	20
Adenocarcinoma	4
Clinical stage (UICC 7th)	
I	7
II	4
III	13
T stage	
1	8
2	2
3	14
N stage	
0	12
1	5
2	7
Tumor location	
Middle third	13
Lower third	11
Operative procedure	
2-field	5
3-field	19
Surgery	
Open	5
VATS	19
Operative time (min)^a^	412 ± 63
Postoperative complications (Clavien–Dindo Grade ≥ 2)	
Anastomotic leakage	1
Pneumonia	4
Liver dysfunction	2
Chylothorax	1
Surgical site infection	1
Peritonitis	1
Venous thromboembolism	1
Postoperative hospital stay (days)^a^	14.6 ± 3.4

*VATS* video-assisted thoracic surgery

^a^Mean ± standard deviation

### Feasibility

**Fig. 1:**
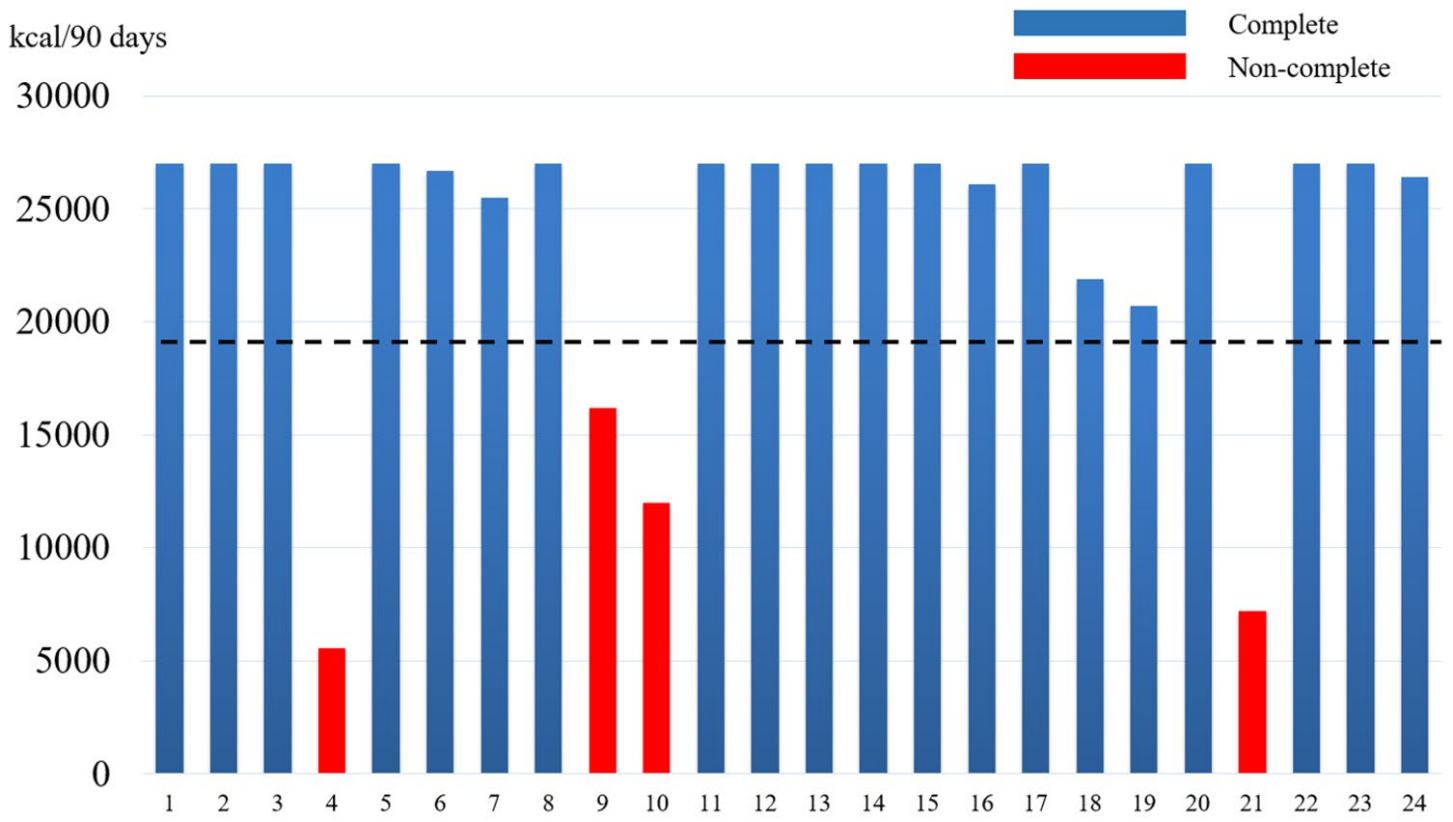
Nutrition provided by STN that was administered over 90 days in 24 patients. A total of 20 patients achieved 70% or more of the planned nutrient intake. Dotted line indicates the level of completion

A total of 20 of the patients were administered 70% or more of the planned nutrient, with a ratio of patients who completed the nutritional intervention of 83.3% (90% CI 65.82–94.1%) (Fig. 1). Since the lower confidence interval was above 60% of the threshold, we could evaluate that the planned nutrition intervention was feasible.

There were 14 patients who were administered the total amount of the planned nutrient. The completion rate was 88.9% for males and 66.7% for females, and it was 75.0% for patients with advanced disease (stage II/III disease) and 100% for patients with stage I disease.

There were 4 patients (16.7%) who could not complete the nutritional intervention. The reasons for the discontinuation were as follows: 3 patients discontinued nutritional intervention due to patient choice, and 1 patient was found to have hemodyscrasia during the intervention. One patient removed the tube by himself after administering only 76% of the planned nutrient.

### Safety

All 24 patients could be assessed for AEs during the intervention. There was no grade 3/4 AE. Grade 2 dermatitis caused by the tape used to fix the tube in place occurred in 1 patient (4.2%). Grade 1 diarrhea occurred in 1 patient (1.2%). Both AEs improved within 1–2 months. These 2 patients with AEs were able to complete the nutritional intervention.

### Changes of body weight and skeletal muscle

The data collected at each point for secondary factors such as nutritional parameters of body weight, body mass index, serum albumin and serum transthyretin, are shown in Table 2. Data could not be collected due to recurrence in 2 patients and development of myelodysplastic syndrome in 1 patient during the course. The data of each sample were not significantly different than a normal population statistically.

**Table 2:** Changes of nutritional parameters and skeletal muscle area

	Baseline	3 months (Just after intervention)	6 months
	(*n* = 24)	(*n* = 23)	(*n* = 21)
Body weight (kg)	57.3 ± 11.6	52.6 ± 8.0	51.8 ± 7.4
BMI (kg/m^2^)	21.1 ± 2.6	20.0 ± 2.2	19.6 ± 2.2
Serum albumin (g/dL)	4.0 ± 0.4	4.1 ± 0.3	4.2 ± 0.2
Serum transthyretin (mg/dL)	27.6 ± 6.7	21.0 ± 3.6	23.3 ± 4.2
Serum C-reactive protein (mg/dL)	0.2 ± 0.4	0.3 ± 1.1	0.1 ± 0.2
Skeletal muscle area (cm^2^)	58.9 ± 14.3	57.4 ± 18.6	55.1 ± 17.3

Data are presented as mean ± standard deviation

**Fig. 2:**
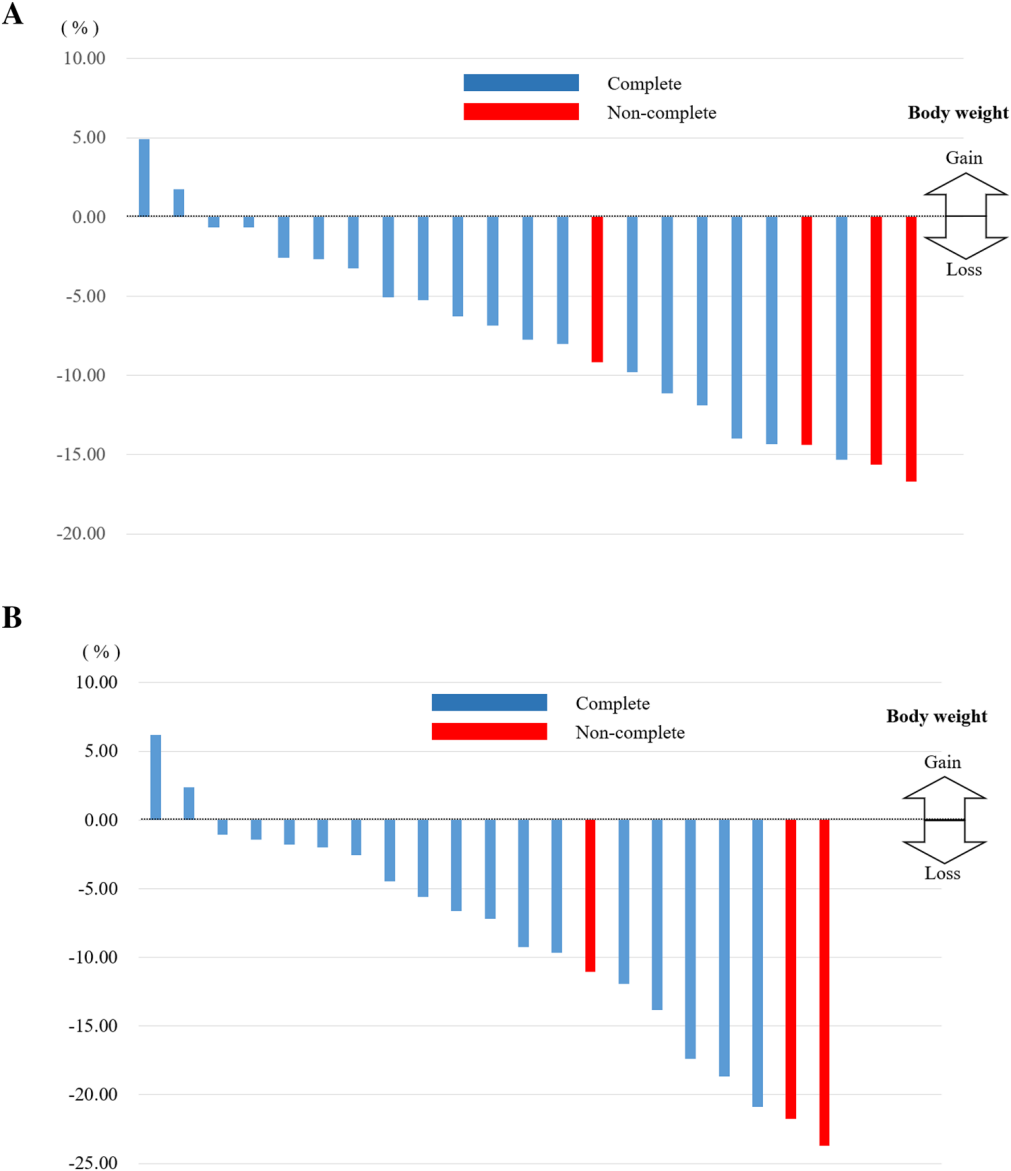
Body weight changes at 3 months (**a**) and 6 months (**b**) after surgery are shown in a waterfall plot

Mean preoperative body weight was 57.3 ± 11.6 kg, while body weight was 52.6 ± 8.0 kg at 3 months after surgery and 51.8 ± 7.4 kg at 6 months after surgery. Mean body weight change was − 7.6 ± 6.0% at 3 months, and − 8.7 ± 8.3% at 6 months after surgery, with the body weight changes shown in the waterfall plot (Fig. 2). An increased body weight was seen in 2 (8.3%) patients immediately after the nutritional intervention. Decreased body weight of more than a 10% loss was observed in 8 (33.3%) patients, while 3 (12.5%) patients had a loss greater than 20%.

**Fig. 3:**
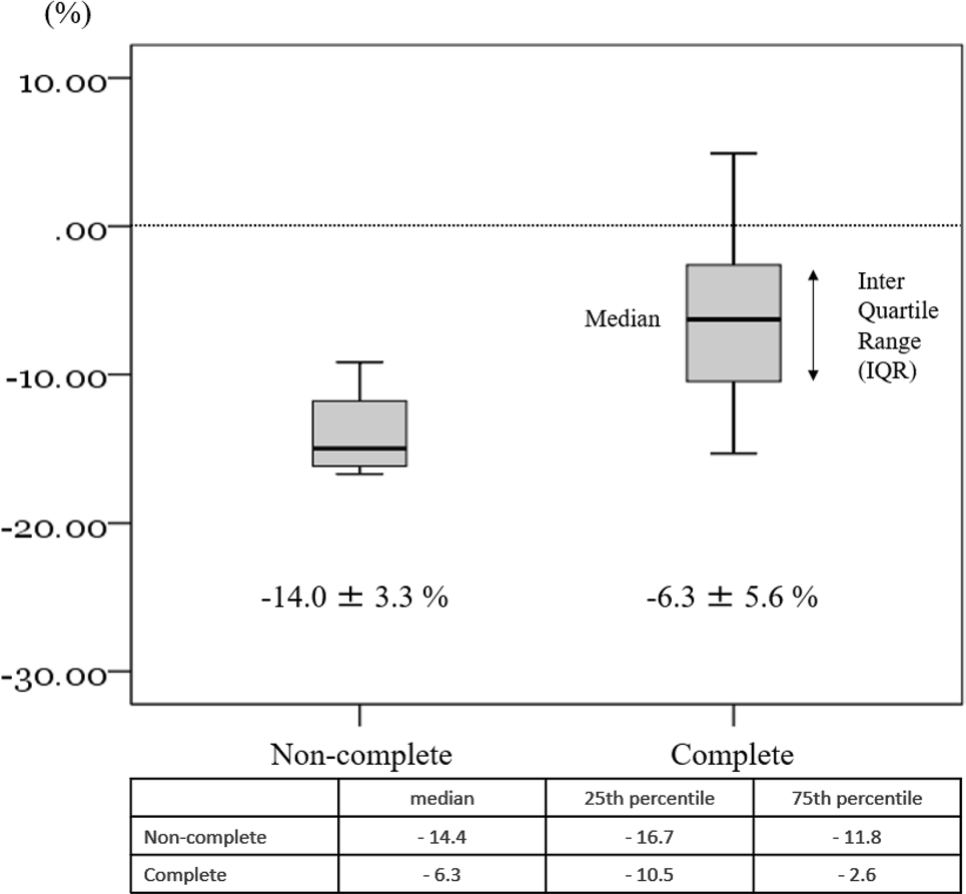
Mean body weight change at 3 months after surgery was − 6.3 ± 5.6% in 19 patients who completed the nutritional intervention, and − 14.0 ± 3.3% in 4 patients who did not complete the nutritional intervention

**Fig. 4:**
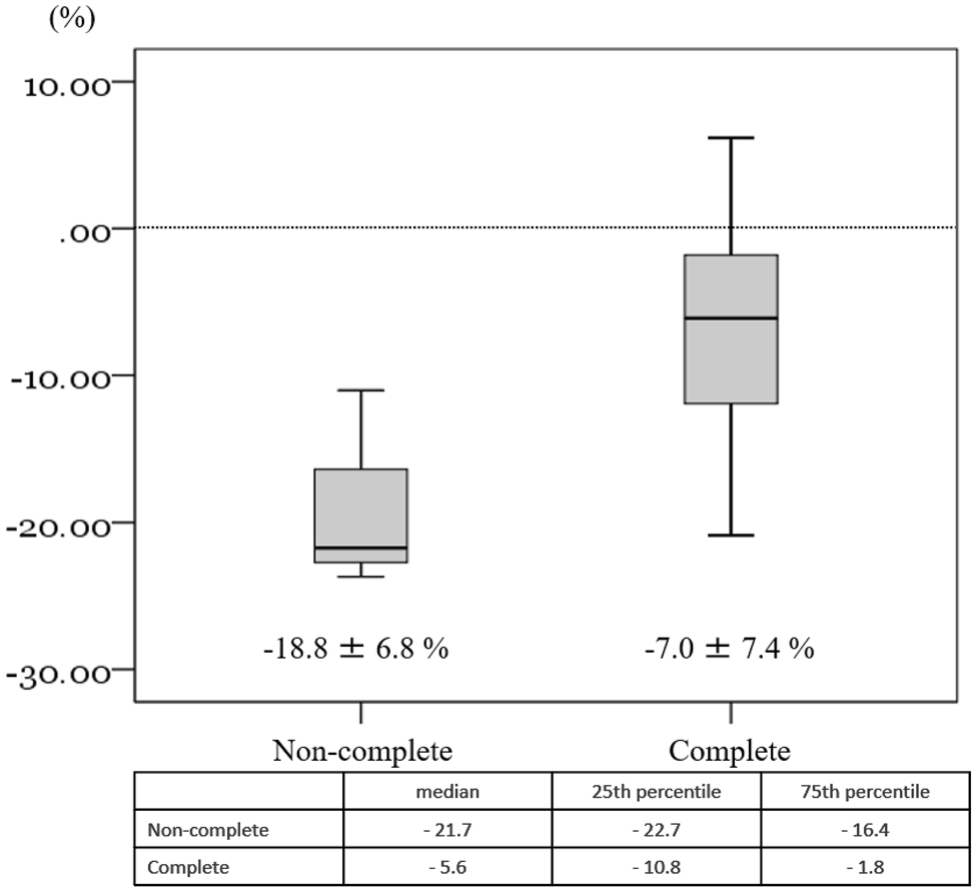
Mean body weight change at 6 months after surgery was − 7.0 ± 7.4% in 18 patients who completed the nutritional intervention, and − 18.8 ± 6.8% in 3 patients who did not complete the nutritional intervention

Mean body weight change at 3 months after surgery was − 6.3 ± 5.6% in 19 patients who completed the nutritional intervention, and − 14.0 ± 3.3% in 4 patients who did not complete the nutritional intervention (Fig. 3). Mean body weight change at 6 months after surgery was − 7.0 ± 7.4% in 18 patients who completed the nutritional intervention, and − 18.8 ± 6.8% in 3 patients who did not complete the nutritional intervention (Fig. 4).

**Fig. 5:**
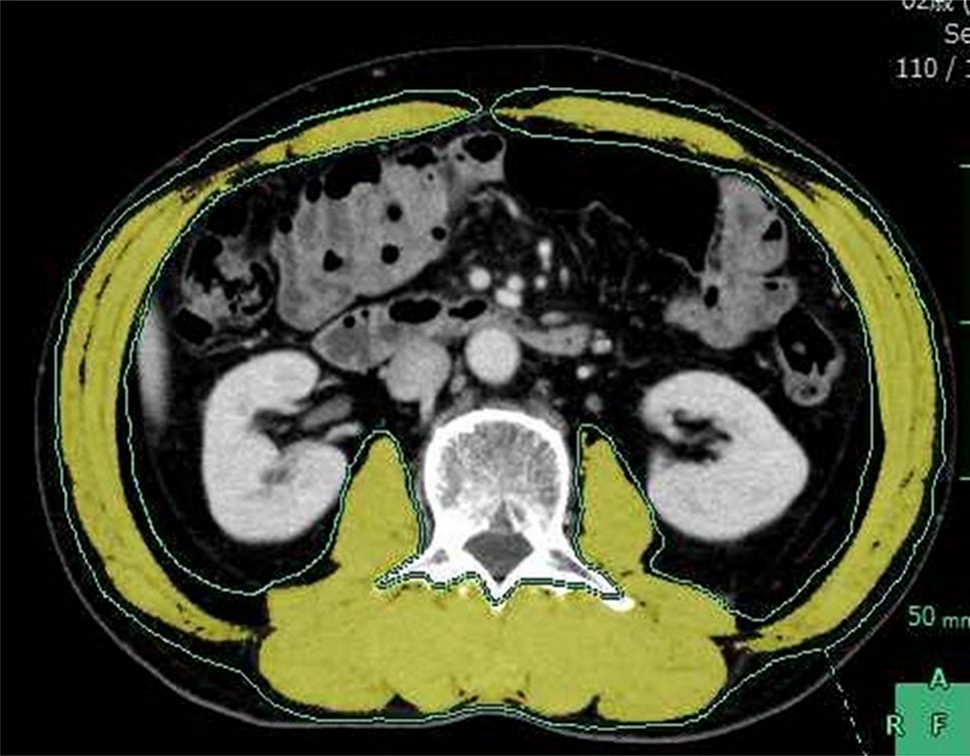
Skeletal muscle in the cross-sectional area at the third lumbar vertebra

**Fig. 6:**
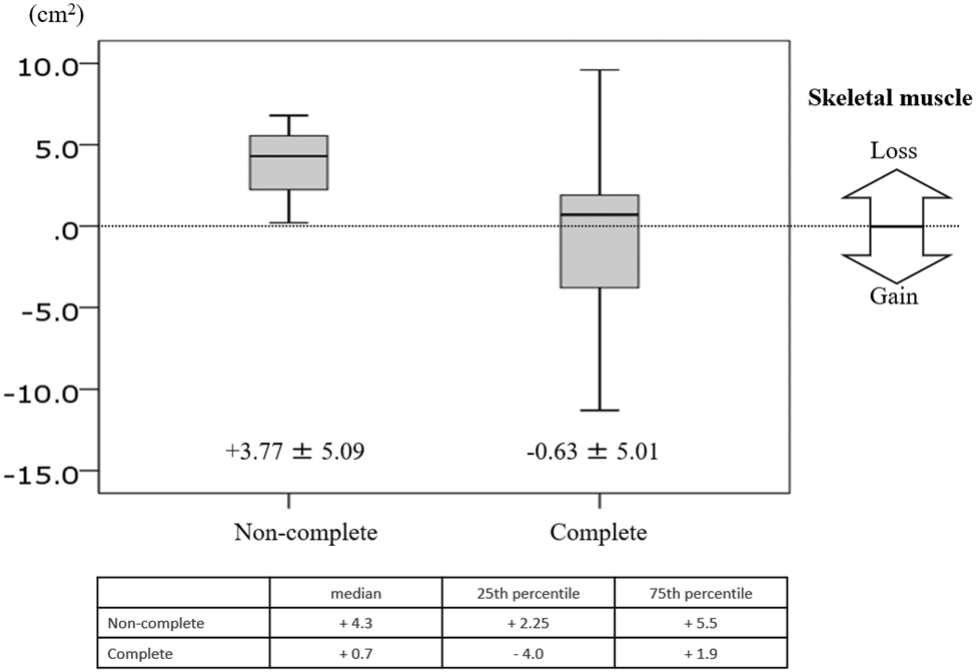
Loss of skeletal muscle in CSA was − 0.63 ± 5.01 cm^2^ in 18 patients who completed the nutritional intervention, and + 3.77 ± 5.09 cm^2^ in 3 patients who did not complete the nutritional intervention

We investigated the correlation between the completion of nutritional intervention, and the decrement in the skeletal muscle in the CSA located at the third lumbar vertebra (Fig. 5) before and at 6 months after surgery. Loss of the skeletal muscle in the CSA was − 0.63 ± 5.01 cm^2^ in 18 patients who completed the nutritional intervention, and + 3.77 ± 5.09 cm^2^ in 3 patients who did not complete the nutritional intervention (Fig. 6).

## Discussion

As previously mentioned, patients who undergo surgical resection of upper gastrointestinal malignancies, including esophageal cancer, are known to have a significant decrease in body weight and QOL due to long-term inadequate oral intake [8, 9]. Nevertheless, various attempts have been made to counteract these changes. With regard to existing enteral nutrients, more than 80% of patients after surgery who have difficulty eating a meal have been reported to exhibit improvement in conjunction with various nutritional indicators [10, 11]. Therefore, while it can be expected that nutritional intervention might be able to help avoid significant weight loss and the subsequent loss of QOL, performing continuous nutritional intervention at home is often difficult due to a variety of factors.

In general, when providing nutritional support, the first thing to do is food fortification, mainly with regard to meals, and often in conjunction with nutritional guidance at the same time. However, this alone does not always control weight loss in patients after surgical resection for esophageal cancer.

In contrast, there have been reports that several beneficial effects can occur after the introduction of ONS [10, 11]. However, some patients refrain from the intake of regular meals when taking postoperative ONS. Thus, given that ONS is considered to be an additional oral ingestion of food with a special medical purpose in conjunction with a normal diet, the benefits of ONS may not occur in some cases.

In this study, we evaluated tube feeding in enteral nutrition. It is thought that regular meals will not be affected as much as that normally observed with ONS. However, on the other hand, there have been a few studies that have examined whether it is practical or even possible to administer the prescribed amount of food intake due to the problems associated with educating and helping patients to understand the issues involved with this procedure. Therefore, we evaluated the feasibility of continuing STN for 3 months after esophageal cancer surgery using enteral nutritional supplements, by examining the changes in the body weight, lean body mass, and muscle mass and the use of these factors in actual clinical practice. Our results demonstrated that the routine use of 90-day home STN after hospital discharge for esophagectomy patients is both feasible and acceptable.

It has been previously reported that home enteral nutrition after discharge from esophageal cancer surgery improves not only the undernutrition but also the QOL [12, 13]. Generally, nutritional administration via a jejunostomy, along with intermittent administration, is not recommended in these patients. Furthermore, enteral administration is often performed using a pump at night. Initially, it was assumed that there would be two major AEs: the appearance of digestive symptoms due to the administration of nutritional supplements, and the troubles caused by tube management. However, the former AE of gastrointestinal symptoms was controllable, and the AE that prevented completion was tube-related problems. Our current study confirmed that intermittent administration of small doses during the day without a pump can be managed in the patient without encountering any major AEs. Based on our findings, we believe that the simple method of using a syringe to administer nutrients without having to learn how to handle a pump is easy for many patients to understand and thus, can be introduced.

Although this study is an initial feasibility test, body weight loss and changes in muscle mass, which are secondary factors, were suppressed as compared to that reported in previous studies. Patients in our study who completed the nutritional intervention had a weight loss rate of –6.3% at 3 months. However, considering that the rate of weight loss in the patients who could not complete the nutritional intervention for various reasons was –14.0%, this suggests that the addition of a 300 kcal daily nutritional supplement may be sufficiently effective in helping to suppress weight loss. Also of note is the finding that the weight loss rate at 6 months after completing the nutritional intervention was reduced in those patients who were able to successfully complete this nutritional intervention. This suggests that nutritional intervention during the first 3 months, when weight loss is significant, might be able to affect subsequent changes in both the weight and QOL.

We also examined the decrease in skeletal muscle, such as sarcopenia, which has recently attracted attention. Enevo™ is a nutritional supplement that contains relatively large amounts of branched chain amino acids **(**BCAA), which can be expected to be effective in maintaining skeletal muscle through nutritional intervention. In adjuvant chemotherapy for gastric cancer, weight loss and skeletal muscle loss have been reported to affect treatment completion [14–16]. In the esophageal cancer patients, recurrence within 1 or 2 years after surgery accounts for a large percentage of the 85% of the patients in which some kind of therapeutic intervention was required to be performed [17–19]. Suppressing weight loss and maintaining skeletal muscles as much as possible within 1 year after surgery may temporarily expand the range of possible treatment options, in addition to potentially increasing the rate of treatment completion, thereby improving the prognosis.

This study has several limitations worth noting. First, this was a single-center, prospective, and single-arm study and was designed with the purpose of verifying the feasibility of STN. Therefore, the number of cases was set to be very small. Although the data of each variable were not significantly different than a normal population statistically, there were variations and the shape of the box plots were also distorted due to the small number of samples. Based on the results of this study, new clinical trials are needed to evaluate the efficacy and effectiveness of this method of nutritional intervention. It should be avoided to conclude that the analysis the secondary factors including small number of patients in this study could suppress body weight loss and skeletal muscle loss. However, in cases where nutritional interventions have been completed, it is considered possible to expect the impact on nutritional factors. Second, since this study was aimed at patients who understood the meaning and purpose of nutritional intervention and agreed to the study, we could not refer to patients who were not active in nutritional intervention. It is essential to show the importance of nutritional intervention as an evidence to such patients, especially those who have severe weight loss and decreased physical activity. Third, the study did not consider the oral intake of patients. This was because we evaluated whether the STN nutritional intervention after oral intake within the patients’ possible range was feasible or not. When evaluating the effectiveness of nutritional interventions in the future, it is important to consider the nutritional intake to include total energy (kcal/d) and protein intake (g/day), contribution of oral intake (food, fluids), oral nutritional supplements.

When considering weight loss and the maintenance of skeletal muscle, anamorelin, which is a potent agonist of the ghrelin receptor, has attracted attention as a different approach for use in lung and gastrointestinal cancers, including stomach cancer [20, 21]. In addition to improving appetite, this is also expected to help increase the weight gain and lean body mass. In contrast, it has also been reported that maintaining or increasing skeletal muscle mass does not always lead to an improvement in physical activity associated with these changes. Even in this study, it was not clear whether nutritional intervention contributed to the weight loss and maintenance of skeletal muscle, in conjunction with the improvement of the physical activity. Therefore, it may be necessary to not only use nutritional intervention but also include rehabilitation in the patient’s recovery program. In these patients, both nutrition therapy and exercise therapy are associated with achieving overall good long-term results. Thus, while it is important to control the weight loss, we believe that it is more important to maintain the patient's physical activity and return to society, thereby helping to improve their QOL.

In subsequent studies, we will need to assess the association between the use of STN and the improvement in both the nutritional status and physical activity.
